# Single-cell analysis of autophagy activity in normal and de novo transformed human mammary cells

**DOI:** 10.1038/s41598-020-77347-w

**Published:** 2020-11-20

**Authors:** Sylvain Lefort, Sneha Balani, Davide Pellacani, Boris Guyot, Sharon M. Gorski, Véronique Maguer-Satta, Connie J. Eaves

**Affiliations:** 1 Terry Fox Laboratory, BC Cancer Agency, 675 West 10th Avenue, Vancouver, BC V5Z 1L3 Canada; 2 Centre de Recherche en Cancérologie de LyonInserm U1052-CNRS UMR5286, Centre Léon Bérard, Lyon, France; 3 Canada’s Michael Smith Genome Sciences Centre, BC Cancer Agency, Vancouver, BC Canada

**Keywords:** Autophagy, Mammary stem cells, Tumour heterogeneity

## Abstract

Assessment of autophagy activity has historically been limited to investigations of fixed tissue or bulk cell populations. To address questions of heterogeneity and relate measurements to functional properties of viable cells isolated from primary tissue, we created a lentiviral (RFP-GFP-MAP1LC3B) vector that allows the autophagosome and autolysosome content of transduced cells to be monitored at the single-cell level. Use of this strategy to analyze purified subsets of normal human mammary cells showed that both the luminal progenitor-containing (LP) subset and the basal cells (BCs) display highly variable but overall similar autophagic flux activity despite differences suggested by measurements of the proteins responsible (i.e., LC3B, ATG7 and BECLIN1) in bulk lysates. Autophagosome content was also highly variable in the clonogenic cells within both the LPs and BCs, but the proliferative response of the BCs was more sensitive to autophagy inhibition. In addition, use of this vector showed cells with the lowest autophagosome content elicited the fastest tumor growth in 2 different models of human mammary tumorigenesis. These results illustrate the utility of this vector to define differences in the autophagy properties of individual cells in primary tissue and couple these with their responses to proliferative and oncogenic stimuli.

## Introduction

Autophagy refers collectively to a process that delivers proteins, organelles, and other cytoplasmic components to the lysosome for destruction and subsequent recycling^1–3^. This process involves the creation of organelles called autophagosomes that then fuse with lysosomes through a series of steps mediated by a group of highly conserved gene products collectively referred to as AuTophaGy-related (ATG) proteins. These include LC3B (MAP1LC3B, microtubule-associated protein 1 light chain 3 beta) that is referred to as LC3B-I when present in a processed cytosolic form, but as LC3B-II when it becomes associated with autophagosomes via a phosphatidylethanolamine connection^4^. Assessments of autophagy activity in mammalian cells have historically relied on measurements of LC3B-II protein levels, or of LC3B-positive vesicles, as indicators of the cellular content of autophagosomes^5^. Because P62 is incorporated into the completed autophagosome and degraded in autolysosomes, levels of P62 have served as an index of autophagy-mediated degradation activity^6–8^. BECLIN1 (BECN1) is also relevant to the autophagy process through its ability to modulate autophagy activity^5^. During nucleation, the ATG proteins are hierarchically recruited to the phagophore assembly site by a complex integrated by BECLIN 1 and hVps34/class III PHOSPHATIDYLINOSITOL 3-KINASE (PI3K). The elongation of the autophagosomal membrane is controlled by 2 ubiquitin-like protein conjugation systems: ATG12-ATG5 and ATG8/LC3^9^.

Autophagy has a well-documented role in many aspects of normal mammary tissue homeostasis, but in human cells, has been limited to evidence of a general activation in the immortalized non-tumorigenic MCF10A human mammary cell line and histologic analysis of human mammary tissue^10,11^. In malignant cells, multiple roles of autophagy, both promoting and suppressing the genesis and growth of the cells has been extensively studied^12–14^. These include protection against the mutagenic effects of reactive oxygen species that then promote transformation, as well as the promotion of survival of fully malignant cells experiencing hypoxia during the initiation of tumor formation in vivo before vascularization has occurred. In various mouse models, decreased autophagy associated with a loss of *Becn1/ATG6* (the gene encoding BECLIN1) promoted the growth of precancerous cells and tumor formation and similar results (reduced BECLIN1 levels) in human cells have been inferred from comparisons of human breast carcinomas and normal human breast tissue^15–18^. Overexpression of BECLIN1 in the MCF7 human breast cancer cell line reduced the proliferative activity of these cells in vitro and decreased their tumorigenic activity in vivo^15^. However, studies of MMTV-PyMT and *Palb2*-deficient mouse cells identified a tumor-promoting role of autophagy in both of these oncogene-driven breast cancer models^19,20^.

Given this complex picture and a paucity of information about the control and role of autophagy in primary sources of normal human mammary cells before and after exposure to an oncogenic stimulus, we initiated a study to examine these questions directly. For this, we isolated different purified subsets of normal viable human mammary cells from primary reduction mammoplasty tissue samples^21^ and examined them before and after initiation of their malignant transformation by forced expression of a lentivirally introduced *KRAS*^*G12D*^ cDNA^22^. To enable differences in the content of LC3B-I and LC3B-II of different human mammary cell phenotypes to be coupled directly with their functional properties at a single cell level, we created a lentiviral vector encoding the widely used RFP-GFP-MAP1LC3B construct^5,23^, and then used it to assess transduced subsets of normal and *KRAS*^*G12D*^ co-transduced human mammary cells. Using this approach, we reveal significant differences in the autophagy activities of normal human mammary cells with luminal and basal features, in their colony-forming cells (CFC) activities, and in their initial responses to induced transformation.

## Results

### Creation of a RFP-GFP-LC3B lentiviral vector enabling analysis of autophagy activity in single viable cells

**Figure 1:**
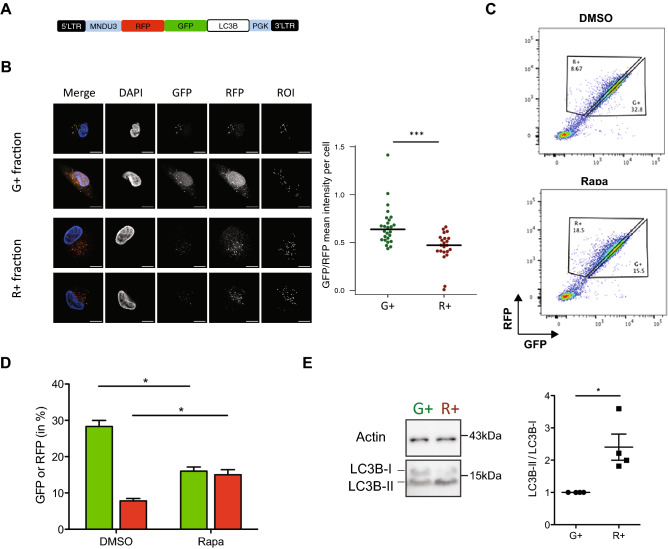
FACS analysis of the autophagy activity in lentiviral *RFP-GFP-LC3B*-transduced human mammary cells. (**A**) Design of the lentiviral RFP-GFP-LC3B reporter construct. Dual RFP^+^GFP^+^ cells indicate the presence of phagophores and autophagosomes. RFP^high^GFP^low^ fluorescence is indicative of the presence of autolysosomes. (**B**) Confocal determination of RFP^+^GFP^+^LC3B puncta in sorted fractions of G^+^ and R^+^ cells within stably *RFP-GFP-LC3B*-transduced MCF10A cells. (**C**,**D**) Effect of a 3-h incubation of *RFP*^*-*^*GFP*^*-*^*LC3B*-transduced MCF10A cells in SF7 medium with 1 µM rapamycin or the equivalent concentration of DMSO (0.5%). Bar graph (**D**) showing the R^+^ and G^+^ cell content of *RFP-GFP-LC3B*-transduced MCF10A cells determined after a 3-h incubation in DMEM/F12 media with 1 µM rapamycin or the equivalent concentration of DMSO (0.5%). (**E**) Validation of the FACS-based quantification of R^+^ and G^+^ MCF10A cells to detect changes in their LC3B-I and LC3B-II protein content determined by WB analysis. Statistical analysis: P values were determined using a paired t-test test (**B**) unpaired t-test (**D**,**E**). *P < 0.05; ***P < 0.001.

To facilitate measurements of the autophagosome content of individual viable cells, we created a lentivirus encoding a RFP-GFP-LC3B tandem construct (Fig. 1A). This construct allows autophagosomes to be seen as fluorescent yellow vesicles because they are positive for both GFP and RFP, and also distinguished from autolysosomes, which display a red fluorescent signal because the GFP signal is quenched in the acidic environment of the lysosomes with which the autophagosome has fused. We then used confocal fluorescence microscopy to examine the content of autophagosome foci in transduced MCF10A cells selected by FACS for their differential expression of RFP (R^+^ cells, autophagy-high) or GFP (G^+^ cells, autophagy-low). The results of these confocal measurements confirmed that the G^+^ cells were predominantly expressing autophagosomes, whereas the R^+^ cells contained more autolysosomes (Fig. 1B), in agreement with previously reported data for cells transfected with the same internal construct^24,25^.

We then exposed transduced, but unselected MCF10A cells to 1 µM rapamycin for three hours and reanalyzed the cells for RFP and GFP expression by FACS at the end of the exposure period (Fig. 1C). This showed rapamycin exposure increased the proportion of R^+^ cells, and diminished the proportion of G^+^ cells, compared to controls (Fig. 1D). Western blot (WB) analysis of the isolated R^+^ and G^+^ cells showed that the control G^+^ cells contained equivalent levels of LC3B-I and LC3B-II, whereas the R^+^ cells contained mostly the LC3B-II form (Fig. 1E). This increase in the ratio of LC3B-II to LC3B-I in the R^+^ cells as compared to the G^+^ cells served to demonstrate the ability of flow cytometric analysis of RFP-GFP-LC3B–transduced cells to reveal induced changes in autophagy activity at the single-cell level.

### Different subsets of normal human mammary cells display similar autophagy flux activity despite different levels of ATG-related proteins

Three major types of human mammary cells; basal cells (BCs), a luminal progenitor-containing subset (LPs) and a more mature luminal cell subset (LCs) can be individually isolated in a viable state directly from enzymatically dissociated normal female reduction mammoplasty tissue by fluorescent-activated cell sorting (FACS) based on their separate or dual expression of two cell surface markers, EpCAM and α6-integrin (recognized by antibodies to CD326 and CD49f, respectively) in combination with antibodies to CD45 and CD31 to remove contaminating hematopoietic cells and endothelial cells, respectively. The remaining non-mammary, stromal cells (SCs) present can also be separately isolated using this strategy as they are negative for all of the above markers^21,26,27^.

**Figure 2:**
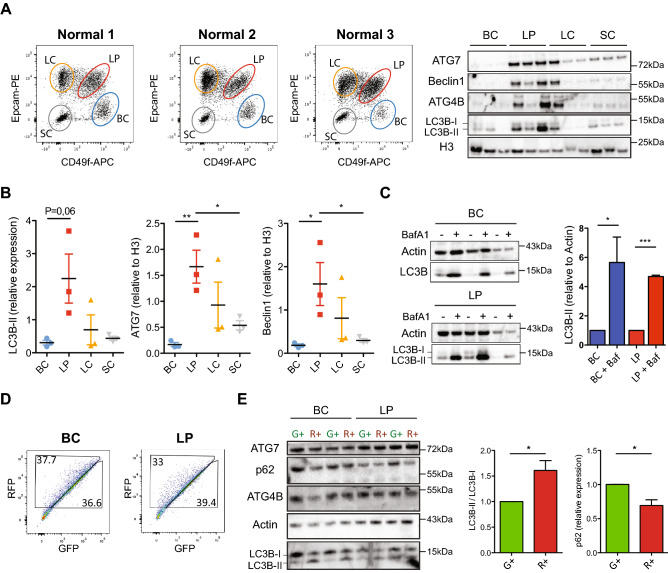
Normal human mammary cell subsets contain different levels of ATG proteins and autophagosomes. (**A**) Representative FACS profiles of human mammary subsets isolated from three normal donors and WBs of their content of different autophagy proteins with H3 as an internal loading control. (**B**) Scatter plots showing the corresponding quantification of human LC3B-II, ATG7 and BECLIN1 normalized to H3 measured independently in subsets isolated from the same three normal samples shown in (**A**). (**C**) Effect of a two-hour exposure of FACS-purified BCs and LPs from three different normal donors (**A**) to 200 nM BafA_1_ (Baf [ +]) or an equivalent concentration of DMSO (0.5%) on their content of LC3B-II (relative to ACTIN). (**D**) Distribution of R^+^ and G^+^ cells in FACS profiles of *RFP-GFP-LC3B*-transduced BCs and LPs analyzed after four days in culture. (**E**) Levels of ATG7, P62, ATG4B, ACTIN and LC3B determined in WBs of the G^+^ and R^+^ cells isolated from the four-day cultures shown in (**D**). Bar graphs show the ratios of LC3B-II/LC3B-I and P62/actin levels in the four subsets of cells analyzed. P-values were calculated using a paired t-test. *P < 0.05, **P < 0.01.

In a first series of analyses, we isolated BCs, LPs, LCs and SCs at high purity (> 96%) from the same donor samples and examined their content of 4 key ATG proteins; ATG7, BECLIN1, LC3B-II and ATG4B by WB analysis. This showed consistently higher levels of both ATG7 and BECLIN1 in the LPs than in the BCs, and intermediate levels of these in the LCs and SCs, with similar trends for LC3B-II and ATG4B (Fig. 2A,B and Supplementary Fig. S1A).

Since inhibition of the autophagic maturation step mediated by fusion of the autophagosomes with lysosomes could lead to an accumulation of both LC3B-II and autophagosomes, we next asked whether the higher levels of ATG proteins in the LPs compared to the BCs might reflect a more active autophagosome generation process in LPs rather than a slower rate of autophagosome-lysosome fusion. To distinguish between these alternatives, we treated purified normal human mammary BCs and LPs in vitro for two hours with 200 nM BafilomycinA_1_ (BafA_1_), an established inhibitor of the autophagosome fusion step^5^. WB comparison of the LC3B-II content in the BafA_1_-treated cells compared to controls showed the same increased levels of LC3B-II in both the treated BCs and LPs (Fig. 2C), indicating that their different levels of LC3B-II was not explained by differences in their rates of autophagosome-lysosome fusion.

We then used the lenti-RFP-GFP-LC3B vector to examine the properties of individual BCs and LPs. FACS analysis was performed on cells maintained in vitro in supportive EGF-containing medium for four days after exposure to the vector (to allow for maximal uptake and expression of the transgene, Fig. 2D). This revealed that a high frequency of gene transfer efficiency to both subsets (~ 70%) was consistently achieved, but the levels of RFP and GFP expression in the individual cells in both were very heterogeneous. WB analyses of FACS-isolated R^+^ cells from both BCs and LPs showed a higher content of LC3B-II relative to LC3B-I than in the matching G^+^ cells isolated from the same samples (Fig. 2E). WB analysis also showed P62 levels to be lower in the isolated R^+^ cells compared to the G^+^ cells, confirming an increased rate of autophagy-mediated protein degradation in the R^+^ cells (Fig. 2E). Importantly, R^+^ cells from both the BC and LP subsets did not contain higher levels of ATG7 or ATG4B, again consistent with primary human mammary R^+^ and G^+^ cells being equally autophagy-competent (Fig. 2C and Supplementary Fig. S1B). On the other hand, the fact that BC and LP became similar in their content of ATG7 and ATG4B after 4 days in vitro (Fig. 2E) suggests that the overall levels of these ATG proteins are differentially affected by the culture conditions used, compared to those operative in cells isolated directly from normal human breast tissue.

In addition, because the autophagy process is known to be a dynamic one, it was of interest to examine its stability in the mammary cells maintained for a more prolonged time in vitro. Analysis of the progeny of R^+^ BCs or LPs maintained for seven days in vitro under the same culture conditions showed they remained R^+^ (Supplementary Fig. S2). In contrast, however, initially G^+^ BCs or LPs produced similar numbers of G^+^ and R^+^ progeny (Supplementary Fig. S2).

### Human mammary cells with progenitor activity require autophagy activity despite variable baseline levels

**Figure 3:**
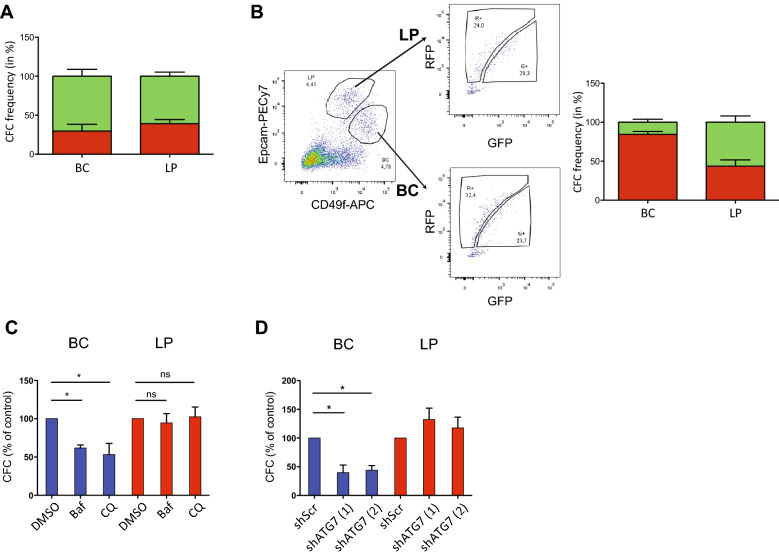
Assessment of CFC frequencies in BC and LP according to their autophagy activity. (**A**) Frequencies of CFCs from the G^+^ and R^+^ cells isolated from four-day cultures of BCs and LPs. Values shown are the mean ± SEM from paired analyses of cells from three normal donors. (**B**) Frequencies of CFCs in the R^+^ and G^+^ fractions of BCs and LPs isolated from collagen gels initially containing 10^5^ *RFP-GFP-LC3B*-transduced BCs and transplanted subcutaneously four weeks previously into female NRG mice. Results are from cells originally isolated from two different normal donors. (**C**) CFC frequencies in FACS-purified BCs and LPs from three different normal donors after incubation at 37 °C for two hours in SF7 medium containing either 200 nM BafA_1_, 10 μM CQ, or an equivalent concentration of DMSO (0.5%). CFC frequencies are expressed as a percent of matched control values. (**D**) Effect of inhibiting ATG7 in BCs and LPs on their CFC activities. Normal BCs and LPs isolated by FACS from three different donors were transduced with pTRIPZ-shScr or shATG7-1 or shATG7-2 vectors, selected for a day with puromycin, and then plated in CFC assays in the presence of doxycycline. CFC activity expressed as a percent of matched control values. P-values were calculated using the Student t-test. *P < 0.05.

To determine whether autophagy is important to the proliferative potential of normal human mammary cells we first transduced FACS-purified BCs and LPs with the RFP-GFP-LC3B vector, and then, four days later, isolated the R^+^ or G^+^ fractions of each and assayed them for clonogenic activity in standard 9-day CFC assays in vitro. The results showed that the CFCs in both the BC and LP subsets were also highly variable in their distribution in their levels of LC3B expression, but overall, appeared slightly enriched in the G^+^ fractions, more prominently in the BCs (P = 0.04, Fig. 3A).

We then asked whether a similar result would be obtained in the BCs and LPs that are regenerated in the bilayered mammary epithelial structures that normal BCs produce in collagen gels transplanted under the kidney capsule of immunodeficient mice^28,29^. Accordingly, mice were transplanted with BCs transduced with the lenti-RFP-GFP-LC3B virus and four weeks later, gels were harvested, single cell suspensions prepared. The R^+^ and G^+^ BCs and LPs in them were then isolated by FACS and the different fractions plated in vitro in the same type of CFC assays. In this case, the results showed that the CFC activity (frequency) in the BCs was higher in the R^+^ subset (higher LC3B-II), but in the LPs, was again not significantly different in the R^+^ and G^+^ fractions (Fig. 3B).

To examine whether BC and/or LP progenitor activities might be dependent on functional autophagy capacity, we then compared the yield of colonies obtained in CFC assays to which 200 nM BafA_1_ or 10 µM chloroquine (CQ) or vehicle was added. In both the BafA_1_- and chloroquine-treated BCs and LPs, blocked fusion of the lysosomes with the autophagosomes was evident as shown by an increase in LC3B-II (Supplementary Fig. S3A). Interestingly, however, both of these autophagy inhibitors significantly and selectively decreased colony yields from the BCs (by 40–50%, P < 0.05), with no detectable effect on colony yields from the LPs (Fig. 3C).

Given the finding that ATG7 levels were similar in the cells generated from BCs and LPs cultured under similar conditions, but at non-limiting cell concentrations (Fig. 2E), and the specific requirement of ATG7 for autophagic function via its role in converting LC3-I to LC3-II^30,31^, we also compared the effect of specifically inhibiting the expression of ATG7 on BC and LP clonogenic activity. For this, we transduced freshly isolated BCs and LPs with either of 2 inducible *shATG7*-puromycin-encoding or a control lentiviral vector and then selected the transduced cells for 2 days in puromycin prior to plating the selected cells in CFC assays. In presence of doxycycline, the BCs transduced with the shATG7 vector showed a 50–60% reduction in the number (and size) of colonies produced compared to controls, whereas the clonogenic activity of the similarly transduced LPs was not affected (Fig. 3D and Supplementary Fig. S3B-C). Taken together, these results show that the clonogenic activity of the phenotypically and functionally distinct BCs and LPs of the normal human mammary gland contain different levels of ATG proteins and respond differently to autophagy inhibition.

### Autophagy levels distinguish human mammary cells with heterogeneous tumor-initiating activities

**Figure 4:**
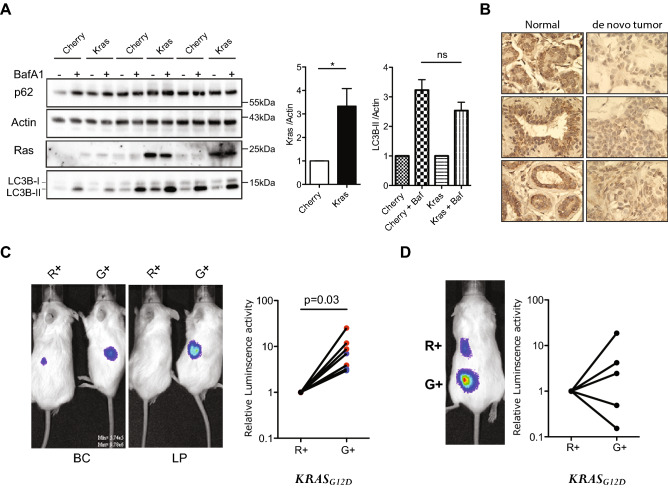
Autophagy differences correlate with initial but not established tumorigenic activity. (**A**) Comparison of the effect of BafA_1_ on LC3B-II levels (relative to ACTIN) in control- and *KRAS*^*G12D*^-transduced human BCs or LPs cells assessed after another three days in vitro. Cells were incubated with 200 nM BafA1 for 2 h and then protein levels determined by WB analysis. Results shown are the mean ± SEM from experiments with cells from three normal individuals (BCs or LPs). (**B**) Representative views of immunostained LC3B in normal donor tissue and matching *KRAS*^*G12D*^-induced de novo tumors. (**C**) Representative pictures of bioluminescent signals measured in mice 2 weeks after the mice were injected subcutaneously with *KRAS*^*G12D*^-transduced G^+^ or R^+^ cells (left panels). The right panel shows the in vivo signals measured in all mice with the data for the G^+^ cells normalized to the matching R^+^ cell data (data for BC-derived cells shown in blue, n = 3, and for LP-derived cells in red, n = 5). P values were calculated using the paired Student t-test. Images were taken with Xenogen IVIS Lumina system and analyzed with Living Image version 3.0 software. (**D**) Similar bioluminescence data for secondary transplants of cells from 5 of the primary transplants of *KRAS*^*G*^^12^^*D*^-transduced cells shown in (**C**).

We next sought to determine whether changes in autophagy components or activity occur during the initiation of human mammary cell transformation. For this, we analyzed the cells in tumors produced rapidly from normal human primary mammary cells transduced with a *KRAS*^*G12D*^-encoding vector and then transplanted into female immunodeficient mice^22^. In an initial set of experiments, we examined the effect of a two-hour exposure of *KRAS*^*G12D*^- or control vector-transduced BCs and LPs to 200 nM BafA_1_ on the expression of LC3BI and LC3B-II in the cells present after another three days in vitro. WB analysis of the cultured *KRAS*^*G12D*^-transduced BCs or LPs exposed to 200 nM BafA_1_ showed an equivalently increased level of expression of LC3BI and LC3B-II compared to the corresponding cultured subsets of control cells (Fig. 4A).

We then used IHC to determine how LC3B levels might be altered in the early transformed progeny of *KRAS*^*G12D*^-transduced cells that are initiating tumor formation two weeks after their transplantation into immunodeficient female mice^22^. Comparison of the LC3B levels in the normal tissue from which the transduced cells had originally been isolated showed the cells in the nascent tumors contained less LC3B (Fig. 4B and Supplementary Fig. S4A).

We then designed an experiment to investigate whether the initial autophagy status of the cells would affect their susceptibility to *KRAS*^*G12D*^-initiated transformation. For this, we first transduced the cells with the lenti-RFP-GFP-LC3B virus, and then four days later, separately isolated their R^+^ and G^+^ derivatives. Equivalent numbers of each of these four cell populations (R^+^ BCs and LPs, and G^+^ BCs and LPs) were then transduced independently with the *KRAS*^*G12D*^-virus and the cells then immediately transplanted into mice. Subsequent bioluminescent tracking of their rates of tumor formation showed the progeny of the G^+^ cells were already growing more rapidly than the R^+^ cells by 2 weeks post-transplant (Fig. 4C), despite a similar efficiency of *KRAS*^*G12D*^ transduction of the initial G^+^ and R^+^ cells (Supplementary Fig. S4B). The more rapid growth of the *KRAS*^*G12D*^-transduced G^+^ cells was confirmed macroscopically using an EVOS fluorescent imaging microscope to size the tumors harvested another 2 weeks later; i.e. 4 weeks post-transplant (Supplementary Figure S4C).

To determine if the initial growth advantage displayed by the *KRAS*^*G12D*^-transduced cells with low autophagy would be perpetuated, 4-week primary tumors initiated from G + cells were dissociated, again sorted into R^+^ and G^+^ phenotypes and the same numbers of each were then transplanted into secondary female mice (5000–30,000/mouse). However, in this case, the rate of secondary tumor growth proved to be the same regardless of the autophagy activity (R^+^ or G^+^ phenotype) of the primary tumor cells from which the secondary tumors were generated (Fig. 4D). Thus the greater primary tumorigenic activity of the *KRAS*^*G12D*^-transduced G + cells isolated directly from human mammary tissue (Fig. 4C) was not sustained.

**Figure 5:**
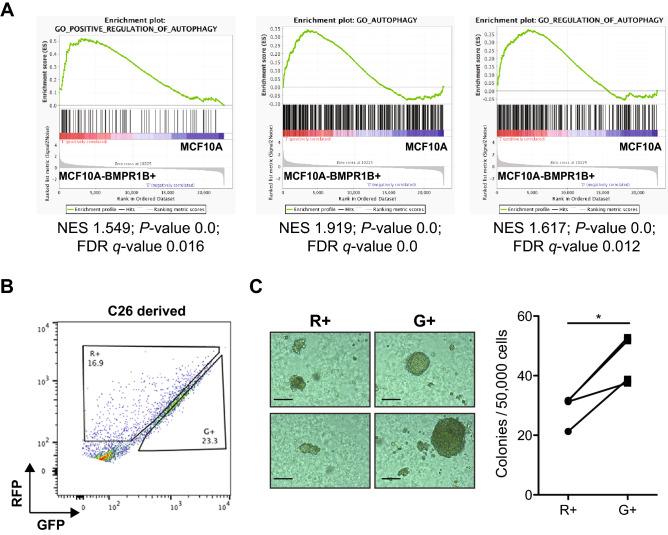
Initial increased tumorigenic activity in low-autophagy compartments is oncogene-independent. (**A**) GSEA of transcripts measured in MCF10A-BMPR1B^+^ compared to MCF10A cells. Gene sets shown are Positive regulation of Autophagy, Autophagy, Regulation of Autophagy. (**B**) Distribution of R^+^ and G^+^ cells in FACS profiles of *RFP-GFP-LC3B*–transduced MCF10A-BMPR1B^+^ cells. (**C**) Representatives pictures of soft agar colonies from R^+^ or G^+^ MCF10A-BMPR1B^+^ cells after three more weeks of culture. The scale bar represents 200 μm. Dot plot showing the frequency of soft agar clonogenic activity comparing FACS-selected R^+^ or G^+^ MCF10A-BMPR1B^+^ cells.

To determine whether this correlation would extend beyond the *KRAS*^*G12D*^ oncogenic stimulus, we applied the same strategy to MCF10A-BMPR1B^+^ cells. These cells have been selected for BMPR1B expression and were then further exposed for 8 weeks to a combination of BMP2 and IL6. MCF10A-BMPR1B^+^ cells are tumorigenic in immunocompromised mice and able to form colonies efficiently in soft agar in contrast to parental MCF10A cells that have neither of these properties^32^. An initial comparison of the transcriptional profiles of the parental MCF10A cells with the MCF10A-BMPR1B^+^ showed the latter display increased autophagy regulatory pathways (Fig. 5A), suggesting the pathways activated by BMP2 and IL6 might be involved in the early steps of transformation. Stable RFP-GFP-LC3B MCF10A-BMPR1B^+^ cells were then generated, and R^+^ or G^+^ fractions isolated by FACS (Fig. 5B). Similar to the response of primary human mammary cells transduced with *KRAS*^*G12*^, the G^+^ MCF10A-BMPR1B^+^ BMP2-IL6-transduced cells displayed an increased capacity to form soft agar colonies compared to the R^+^ cells (Fig. 5C), suggesting that a higher propensity for low autophagy cells to become transformed regardless of the mechanism(s) inducing their transformation.

## Discussion

In this study, we present a first single-cell analysis of the different levels of expression and roles of ATG-related proteins and autophagy activity in the different major cell types that constitute a normal tissue and their derivatives undergoing transformation. To make these analyses possible, we created and exploited the use of a lentiviral vector encoding a *RFP-GFP-MAP1LC3B* cDNA^23^ so that measurements of these different properties could be examined and correlated in the same individual cells in the presence or absence of chemical or molecular inhibitors.

Previous studies in mice and cows have shown that autophagy is differentially activated in different normal mammary cell types during alveologenesis^10,11,33^. However, the extent to which these findings apply to the normal human mammary gland was not known. We now show that in humans, LPs, a subset of mammary cells that share some properties of both BCs and LCs contain more ATG7, BECLIN1 and LC3B-II than BCs. Use of the *RFP-GFP-MAP1LC3B* lentiviral vector also revealed extensive heterogeneity in the autophagy status of both the human BC and LP compartments. Our previously reported higher reactive oxygen species (ROS) content and telomere dysfunction characteristic of human LPs^26,27^ is consistent with their display of a greater autophagy activity^16,34^. In addition, during the menstrual cycle, transiently elevated progesterone levels induce a parallel expansion of the LP compartment, leading to the secretion of RANKL^35^ that has been demonstrated to promote autophagosome formation^36^. Further investigation of mammary cells isolated directly from normal donors at different stages of the menstrual cycle, or from postmenopausal women that display different hormonal status, in order to evaluate how autophagy changes in each subset during that process might therefore be of interest. Nevertheless, autophagy appears to be equivalently functional in freshly isolated BCs and LPs, although BCs displayed a higher sensitivity to molecular or chemical autophagy inhibition than LPs. Whether this is caused by their normal interaction in situ with ECM proteins^33^ could also be of future interest to investigate.

We also provide evidence here that autophagy status is relevant to the initial acquisition of tumorigenic potential by primary human mammary cells. Previous reports have shown that altered autophagy is involved in Ras-mediated tumorigenesis in mouse kidney epithelial cells and the immortalized human MCF10A cell line by helping to maintain mitochondrial metabolic function^37^, and tight regulation of ROS^38^. Here we found that forced expression of *KRAS*^*G12D*^ in BCs and LPs, that have different ROS contents, did not initially change the levels of LC3B-II or autophagy flux in them. However, their early transformed progeny generated in xenografted immunodeficient mice contained less LC3B than normal BCs and LPs. Thus, KRAS^G12D^ does not appear to activate autophagy during the early steps of tumorigenesis induced by expression of this gene in primary human mammary cells, in contrast to events elicited in MCF10A cells. This may be related to the differences that exist between the epigenomes and transcriptomes of the different subsets of normal human mammary cells and immortalized lines of breast epithelial cells that have become established in vitro even though they may not show tumorigenic activity^21^.

In the present study, we also showed that primary sources of normal human mammary cells with high levels of autolysosomes and a high autophagy activity in vitro show impaired tumor initiation in response to forced *KRAS*^*G12D*^ expression and provide evidence that this is not unique to KRAS, as evidenced with our results with the BMPR1B + model. Since high autophagy is associated with tumor progression and resistance to treatment^39,40^, these findings add further support to the concept that autophagy modulation can act as a double-edged sword^41^. Moreover, evidence in the literature suggests that breast cancers display some degree of autophagy dependence and that many Triple-Negative Breast Cancer (TNBC) cell lines are particularly sensitive to autophagy inhibition when compared to luminal cells^40,42^, indicating that autophagy dependence in breast cancer may be subtype-dependent.

Application of the *RFP-GFP-MAP1LC3B* lentiviral vector described here to the analysis of autophagy in more advanced breast cancer models such as TNBC or paired primary and metastatic tumor samples could help to further address the consequences on autophagy heterogeneity in tumor progression and treatment response. Indeed, most of the chemotherapeutic agents are known to be good autophagy inducers in different types of cancer^43^, but how clonal subsets that display different autophagy activity might evolve upon treatment is still to be elucidated. Interestingly, in the present study, the growth advantage of initially appearing transformants obtained from primary cells with low autophagy was not seen in the tumors generated in a subsequent passage. However, we have also previously shown that such secondary tumors are largely derived from different cells than those that initiate the primary tumors^22^. Thus, together, our results illustrate the unique insights into normal human mammary gland homeostasis and the genesis of human breast cancers that can be obtained by single-cell analysis of living *RFP-GFP-MAP1LC3B* vector-transduced cells.

## Methods

### Research guidelines and regulations

All research and cell culture procedures were conducted following written consent according to protocols approved by the University of British Columbia Research Ethics Board.. Informed consent for study participation was obtained from all patients and controls. All research was performed in accordance with relevant guidelines and regulations.

### Cells

MCF10A and MCF10A-BMPR1B^+^ cells were maintained in phenol-free DMEM/F12 medium supplemented with 5% horse serum, 10 mg/ml insulin, 0.5 mg/ml hydrocortisone, 100 ng/ml cholera toxin, 20 ng/ml epidermal growth factor (EGF) (all from Sigma), and 1% penicillin/streptomycin (Life Technologies, Waltham, MA, USA). Rapamycin was obtained from Sigma.

Normal reduction mammoplasty tissue was obtained from premenopausal women with informed consent, according to procedures approved by the University of British Columbia Research Ethics Board. This tissue was then treated to obtain organoid-rich pellets, that were then viably cryopreserved^28^. Thawed organoids were rinsed with Hank’s Balanced Salt Solution supplemented with 2% fetal bovine serum (FBS) (HF), and the cells then dissociated in 2.5 mg/ml trypsin with 1 mM EDTA and 5 mg/ml dispase (STEMCELL Technologies, Vancouver, Canada) with 100 μg/ml DNaseI (Sigma, St Louis, MO, USA) and washing with HF between each step. The resulting cell suspension was filtered through a 40 μm mesh and EpCAM^lo^CD49f.^+^ BCs, EpCAM^hi^CD49f.^+^ LPs, EpCAM^hi^CD49f.^−^ LCs and EpCAM^−^CD49f.^−^ SCs isolated from within the CD45^−^CD31^−^ (blood and endothelial) cells by FACS, as described^28^.

### Confocal analysis

Cells were imaged using a Nikon A1plus confocal system and a 60 × objective. One image of a focal plane crossing through the cell nucleus was taken with the pinhole set at 1.2 AU for the laser with the longest wavelength (561 nm). Signals corresponding to the GFP and RFP fluorescence were first thresholded to generate regions of interest corresponding to cytoplasmic LC3B foci and then measured within the same regions. An average ratio of the GFP and RFP signals were then calculated for each cell. P-values are from Mann–Whitney U tests.

### In vitro assays

For all cultures, SF7 medium supplemented with 5% FBS was used. BafA_1_ and CQ were obtained from Sigma. CFC assays were performed by culturing FACS-purified human mammary BCs or LPs at low density in the presence of irradiated 3T3 fibroblasts for 9 days.

For soft agar colony formation, the bottom agar layer was prepared from 1.5% agar (Promega) diluted in an equal volume of 2X culture medium to a final concentration of 0.75%, added to cell culture plates and incubated at room temperature for 30 min. The top agar layer was prepared accordingly at a final concentration of 0.45%. Cells were mixed into the liquid top agar and added on top of the bottom. Cell culture plates were incubated at room temperature for 30 min and covered with medium. Colonies were quantified after 3 weeks of culture.

### Lentiviral constructs and transduction

Sequence-verified lentiviral vectors containing *MNDU3-RFP-GFP-LC3B, MNDU3-PGK–YFP* (or Cherry), *MNDU3-KRAS*^*G12D*^*-PGK–YFP* (or Cherry), *pTRIPZ-shScr, pTRIPZ-shATG7#1*, or *pTRIPZ-shATG7#2* constructs were used to generate concentrated lentiviral preparations containing ~ 10^9^ infectious units per ml^22^. The *MNDU3-RFP-GFP-LC3B* construct was the same as one previously used to detect the autophagosome content of transfected cells by fluorescent microscopy^44^. All cells were transduced by exposing up to 5 × 10^5^ cells in 100 μl of SF7 medium to each virus-containing supernatant at a final dilution of 1:100. For *pTRIPZ-shScr, pTRIPZ-shATG7#1*, or *pTRIPZ-shATG7#2* constructs (Horizon Discovery), puromycin (0.5 μg/ml) was added one day post-transduction to select only cells that had integrated the vector DNA. Then, doxycycline was added during CFC assay. Doxycycline addition lead to the concomitant expression of RFP and the shRNA in the vector.

### WB analysis

Cells were washed with cold PBS and incubated for 15 min at 4 °C with RIPA lysis buffer (30 mM Tris–HCl, pH 7.5, 150 mM NaCl, 10% glycerol, 1% Triton X-100) supplemented with a 1 mM NaF, 1 mM NaVO3 and 1 mM PMSF (all from Sigma). Cell extracts were centrifuged at 13,000 *g* for 10 min at 4 °C. The protein concentration of the supernatant fraction was determined using the Bio-Rad Bradford Protein Assay Kit according to the manufacturer’s instructions. For each sample, an equal amount of total protein was diluted in sample buffer (Invitrogen) and boiled for 5 min. Samples were loaded onto precast (NuPAGE 4–12% gradient polyacrylamide gels; Invitrogen). After electrophoresis, the proteins were transferred to a PVDF transfer membrane. Membranes were then blotted overnight at 4 °C with the appropriate primary antibodies: i.e., anti-ACTIN (sc-1615, 1/10,000, Santa Cruz, Mississauga, Canada), anti-ATG4B (A2981, 1/1,000, Sigma), anti-ATG7 (NB110-55474, 1/1,000, Novus Biologicals, Littleton, CO, USA), anti-BECLIN1 (D40C5, 1/1,000, Cell Signaling Technologies, Leiden, The Netherlands), anti-H3 (12648, 1/10,000, Cell Signaling Technologies), anti-LC3B (3868, 1/1,000, Cell Signaling Technologies), or anti-RAS (3339, 1/1,000, Cell Signaling Technologies). Specific binding of antibodies was detected using appropriately conjugated secondary antibodies, and visualized with SuperSignal West Femto Maximum Sensitivity Substrate (Thermofisher) on a ChemiDoc Gel Imaging system (Bio-rad). Densitometric analyses of immunoblots were performed using ImageJ.

### Xenografts

Virgin 5- to 10-week-old nonobese diabetic-*Rag1*^*-/–*^*IL2Rγc*^*−/−*^ (NRG) mice were used for all xenografting experiments. These mice were bred and maintained in the specific pathogen-free rodent facility of the British Columbia Cancer Research Centre, according to procedures approved by the University of British Columbia Animal Care Committee. The study was carried out in accordance with the guidelines of the Canadian Council of Animal Care (CCAC). Subrenal xenotransplants of normal human mammary cells were performed as previously described^28^. Briefly, human cells were combined with 10^5^ irradiated (50 Gy) C3H 10 T½ fibroblasts in a 25 μl volume of cold pH-neutralized rat tail collagen and placed into the individual wells of a 24-well plate. After the collagen gels had stiffened in a 37 °C incubator for 10 min, warm SF7 medium plus 5% FBS was added for another 50 min. The plates were then transferred to ice and the gels inserted under the kidney capsule through a 2 to 4 mm incision. A slow-release pellet containing 2 mg β-estradiol and 4 mg progesterone (both from Sigma) was inserted subcutaneously in a posterior position. Four weeks after transplantation, mice were euthanized and the gels removed aseptically from the kidneys and dissociated as described above for normal human mammary samples.

To generate tumors, transduced human mammary cells were injected subcutaneously with 50% (v/v) matrigel into mice^22^. To measure tumor bioluminescence from luciferase expression, mice were injected intraperitoneally with 150 mg/kg body weight d-luciferin (Promega, Madison, WI, USA) and 10 min later imaged using a Xenogen IVIS Lumina system with Living Image version 3.0 software (Caliper Life Sciences, Hopkinton, MA, USA). To prepare cell suspensions from tumors, the tissue was minced with a scalpel, incubated at 37 °C in DMEM/Ham’s F12 media, supplemented with 5% FBS and 300 U/ml collagenase and 100 U/ml hyaluronidase for 1 to 2 h with periodic vortexing, washed with HF, and treated with 2.5 mg/ml trypsin with 1 mM EDTA and 5 mg/ml dispase with 100 μg/ml DNaseI. Human cells were sorted after staining with anti-human-specific antibodies directed against EpCAM and CD298 with simultaneous depletion of mouse cells stained with antibodies specifically directed against mouse CD45 and CD31. For secondary tumor generation, in addition to human markers, cells were isolated also gated for positive RFP or GFP fluorescence.

### Immunohistochemical (IHC) staining

Pieces of tumors obtained from mice or normal breast were fixed in 10% buffered formalin (Fisher), washed in 70% ethanol and embedded in paraffin. Sections of paraffin-embedded tissue (3 mm) were first treated with Target Retrieval solution (DAKO) and then a cytomation serum-free protein block (DAKO) followed by staining with a specific antibody to LC3B (0231–100/LC3–5F10 diluted 1:200 from NanoTools, Teningen, Germany)^40^. A secondary mouse antibody conjugated to horseradish peroxidase and treatment with 3,3ʹ-diaminobenzidine (DAB, DAKO (Burlington, Canada) was used to obtain a brown staining of positive reactions. Consistently negative IgG controls using a similarly prepared slide of normal reduction mammoplasty tissue, and consistently positive controls using a slide of MDA-MB-231 cells were included in each staining run.

### RNA analysis

Gene-set enrichment analyses (GSEA) was performed using the “pre-ranked” tool^45^. The input data for the GSEA procedure were the following: (i) a complete table of genes ranked according to the log_2_ transformed FC between two groups of samples, (ii) a mapping file for identifying transcripts in the corresponding platform; and (iii) a catalogue of functional gene sets from the Molecular Signature Database. Default parameters were used. Inclusion gene set size was set between 15 and 500 and the phenotype was permutated 1,000 times.

### Statistical analyses

Values shown are the mean ± SEM unless otherwise specified. Significance was evaluated using the Student t-test unless otherwise specified.

## Supplementary information

Supplementary Information
