# Supplementary material for: Single-cell analysis of autophagy activity in normal and de novo transformed human mammary cells
Source: Sci Rep. 2020 Nov 20;10:20266. doi: 10.1038/s41598-020-77347-w (PMC7679376; doi:10.1038/s41598-020-77347-w)
Supplement: Supplementary file 1 — Supplementary Information [file 41598_2020_77347_MOESM1_ESM.pdf]

Supplemental information

Single-cell analysis of autophagy activity in normal and *de novo* transformed human mammary cells

Sylvain Lefort[1,2,*], Sneha Balani[1], Davide Pellacani[1], Boris Guyot[2], Sharon M Gorski[3], Véronique Maguer-Satta[2], Connie J Eaves[1]

[1] Terry Fox Laboratory, BC Cancer Agency, 675 West 10th Avenue, Vancouver, BC V5Z 1L3, Canada.

[2] Centre de Recherche en Cancérologie de Lyon, Inserm U1052-CNRS UMR5286, Centre Léon Bérard, Lyon, France.

[3] Canada's Michael Smith Genome Sciences Centre, BC Cancer Agency, Vancouver, BC, Canada.

*Correspondence: slefort@bccrc.ca or sylvain.lefort@lyon.unicancer.fr

The authors declare no conflict of interest.

Running title: Autophagy in human mammary progenitors

Keywords: human mammary cells, progenitor heterogeneity, tumor initiation, tandem RFP-GFP-LC3B, breast cancer.

Figure S1. ATG-related proteins levels in human primary mammary sorted or cultured cells. (A) Quantification of the ratios of human ATG4B to H3 protein levels shown in Figure **2A** (WBs) for matched cells from 3 normal individuals. (B) Quantification of the ratios of ATG7/ACTIN and ATG4B/ACTIN levels shown in Figure **2E**. P values were calculated using the Student t-test.

Figure S2. Maintenance of high autophagy status *in vitro*. RFP and GFP intensities in human BCs and LPs transduced with lenti-*RFP-GFP-LC3B* virus after being sorted 4 days later according to their R^+ or G^+ status and then re-analyzed for their R^+ or G^+ status after another 3 days *in vitro*. P-values were calculated using the Student t-test.

Figure S3. Inducible shATG7 efficiency. (A) Levels of ACTIN and LC3B determined in WBs of FACS-purified primary mammary cells from two different normal donors after incubation at 37ºC for two hours in SF7 medium containing either DMSO (0.5%), 100 or 200 nM $BafA_1$, 2 or 20 μM CQ, , or starved in EBSS media. (B) Demonstration by WB of doxycycline-induced ATG7 expression in HeLa cells transduced with pTRIPZ-shScr or shATG7 (1) or shATG7 (2). After puromycin selection, cells were treated for 48h with doxycyclin before harvesting. (C) Photomicrographs of representative colonies produced by human BCs and LPs transduced with pTRIPZ-shScr or shATG7 (1) or shATG7 (2). Scale bar represents 200μm.

Figure S4. Low autophagy status enhances tumor initiation from cells transduced with $KRAS^{G12D}$. Related to Figure 4. (A) Representative views of IgG staining of normal human breast tissue (negative control) and LC3B immunostaining of MDA-MB-231 cells. (B) High efficiency of $KRAS^{G12D}$-YFP transduction of R^+ and G^+ BC cells (blue symbols) and LPs (red symbols) detected in terms of %YFP+ cells measured after another 3 days *in vitro*. (C) Representative pictures of *de novo* tumors generated from R^+ or G^+ cells 4 weeks after transplantation into mice.

Figure S5. Uncropped images for which boxed areas are shown in the published panels of Figures 1, 2 and 4. (A) Corresponding to Figure 1E. (B) Corresponding to Figure 2A. (C) Corresponding to Figure 2C. (D) Corresponding to Figure 2E. (E) Corresponding to Figure 4A.

Figure S1

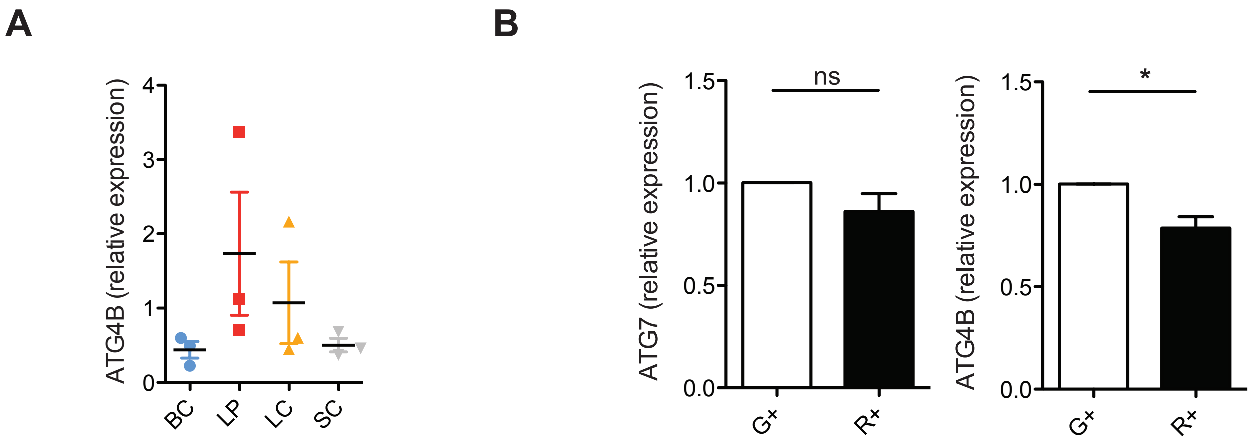
A
B
ATG4B (relative expression)
0
1
2
3
4
BC
LP
LC
SC
ATG7 (relative expression)
ns
*
0.0
0.5
1.0
1.5
G+
R+

Figure S2

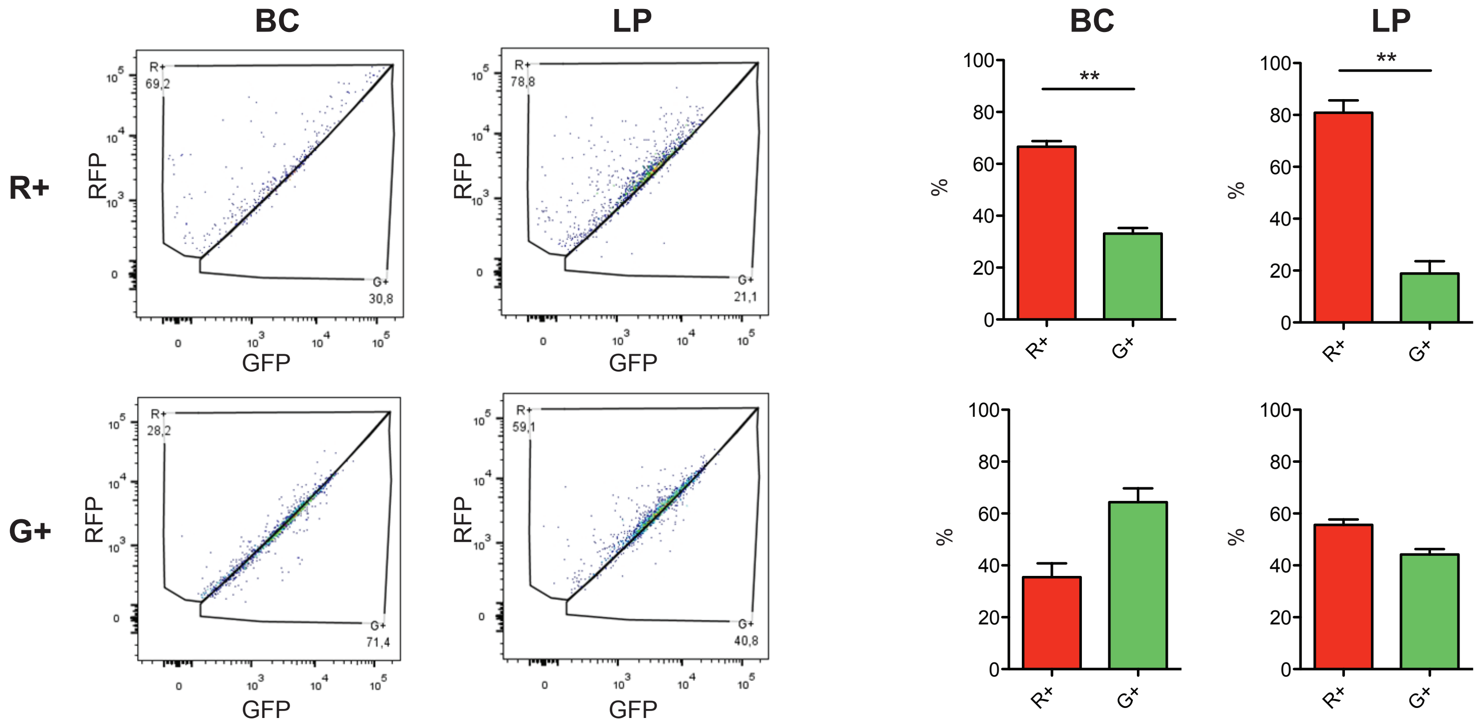

BC
LP
BC
LP
R+
G+
RFP
GFP
R+
69,2
G+
30,8
R+
78,8
G+
21,1
R+
28,2
G+
71,4
R+
59,1
G+
40,8
%
**
R+
G+

Figure S3

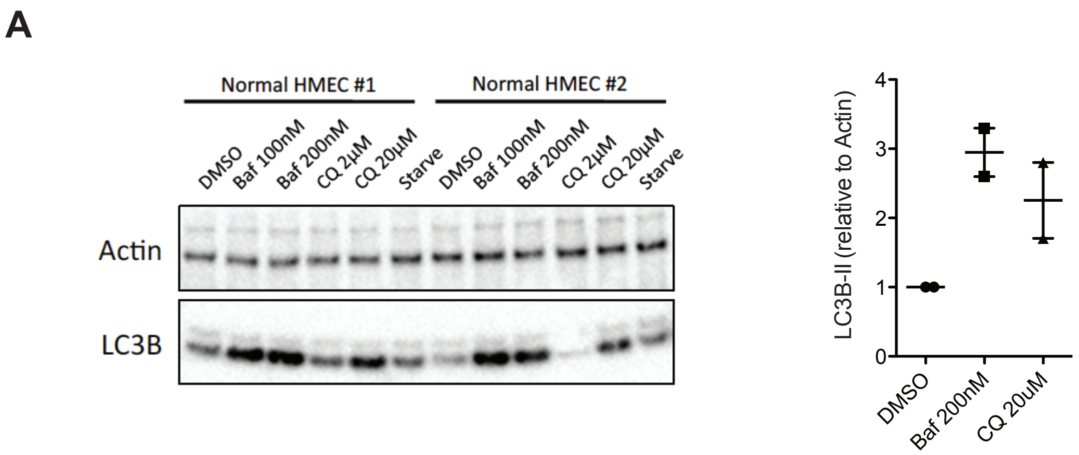
A
Normal HMEC #1
Normal HMEC #2
DMSO
Baf 100nM
Baf 200nM
CQ 2μM
CQ 20μM
Starve
Actin
LC3B
LC3B-II (relative to Actin)
Baf 200nM
CQ 20uM

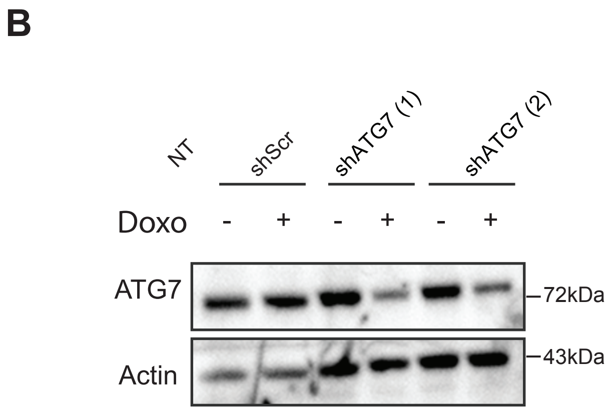
B
NT
shScr
shATG7 (1)
shATG7 (2)
Doxo
- + - + - +
ATG7
Actin
72kDa
43kDa

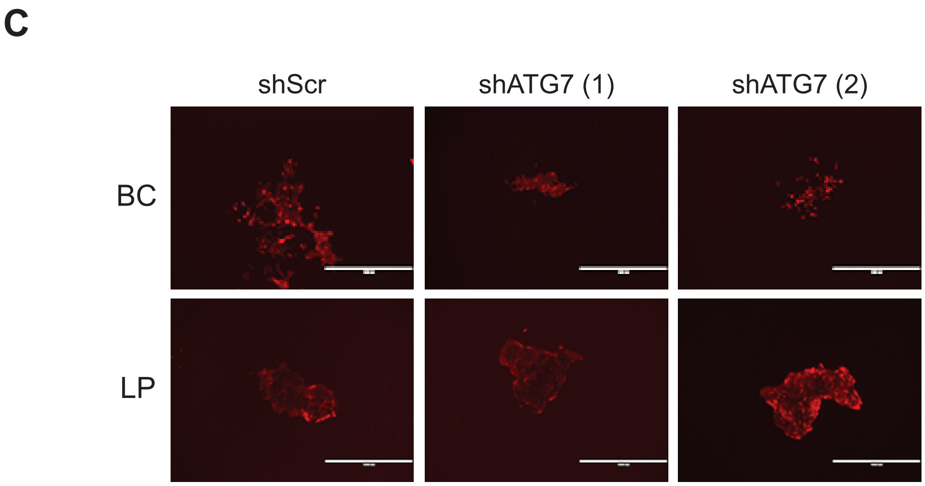
C
shScr
shATG7 (1)
shATG7 (2)
BC
LP

Figure S4

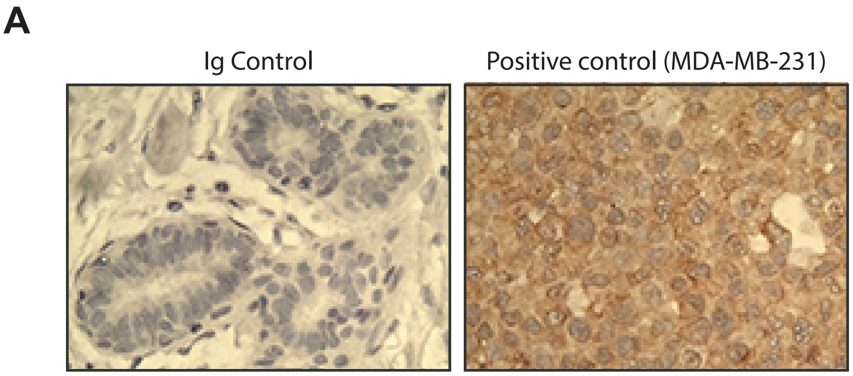
A
Ig Control
Positive control (MDA-MB-231)

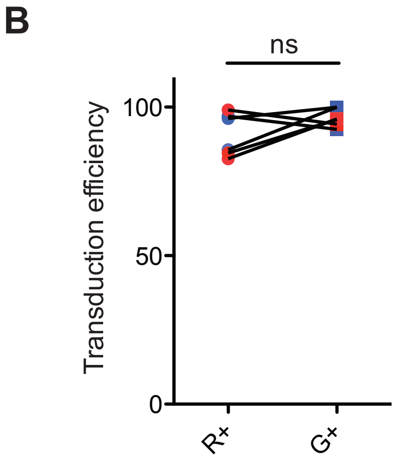
B
ns
Transduction efficiency
100
50
0
R+
G+

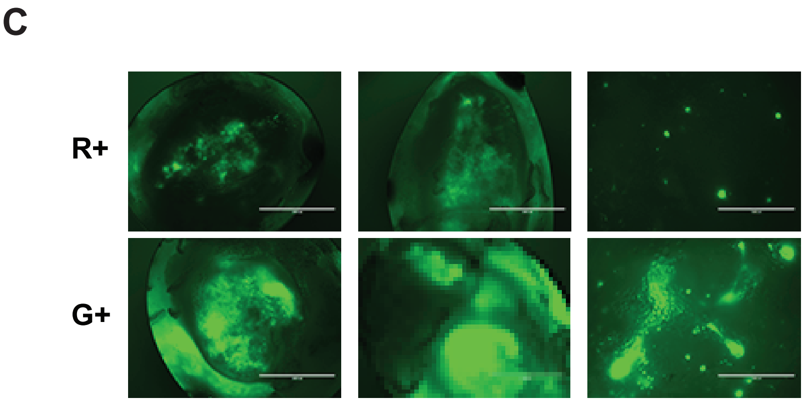
C
R+
G+

Figure S5

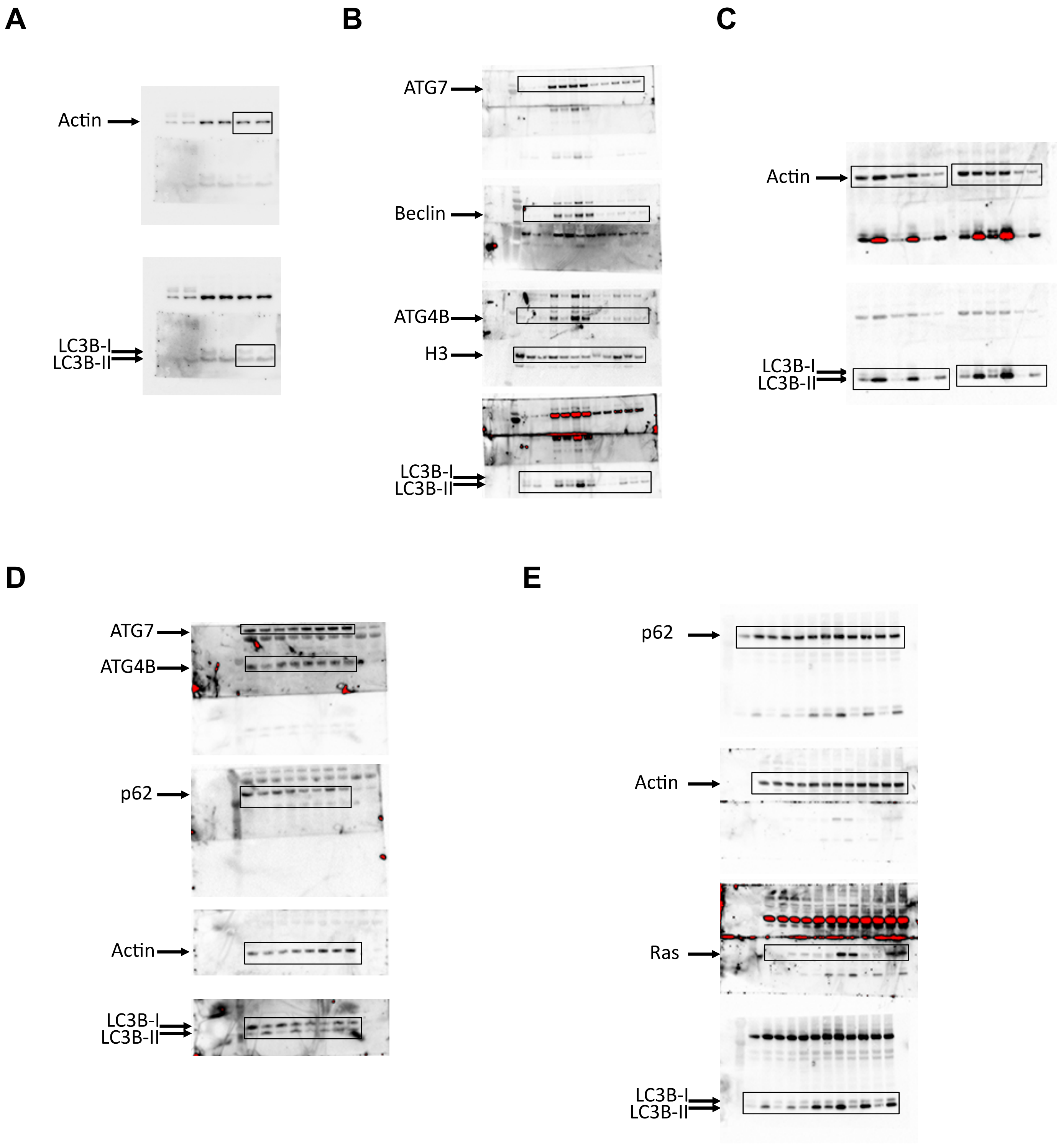
A
Actin
LC3B-I
LC3B-II
B
ATG7
Beclin
ATG4B
H3
LC3B-I
LC3B-II
C
Actin
LC3B-I
LC3B-II
D
ATG7
ATG4B
p62
Actin
LC3B-I
LC3B-II
E
p62
Actin
Ras
LC3B-I
LC3B-II
